# Entropy considerations in improved circuits for a biologically-inspired random pulse computer

**DOI:** 10.1038/s41598-021-04177-9

**Published:** 2022-01-07

**Authors:** Mario Stipčević, Mateja Batelić

**Affiliations:** 1 Photonics and Quantum Optics, Center of Excellence for Advanced Materials and Sensing Devices, Ruđer Bošković Institute, Bijenička cesta 54, 10000 Zagreb, Croatia; 2 Department of Physics, Faculty of Science, University of Zagreb, Bijenička cesta 32, 10000 Zagreb, Croatia

**Keywords:** Information theory and computation, Optical sensors, Electrical and electronic engineering, Mathematics and computing, Optics and photonics

## Abstract

We present five novel or modified circuits intended for building a universal computer based on random pulse computing (RPC) paradigm, a biologically-inspired way of computation in which variable is represented by a frequency of a random pulse train (RPT) rather than by a logic state. For the first time we investigate operation of RPC circuits from the point of entropy. In particular, we introduce entropy budget criterion (EBC) to reliably predict whether it is even possible to create a deterministic circuit for a given mathematical operation and show its relevance to numerical precision of calculations. Based on insights gained from the EBC, unlike in the previous art, where randomness is obtained from electronics noise or a pseudorandom shift register while processing circuitry is deterministic, in our approach both variable generation and signal processing rely on the random flip-flop (RFF) whose randomness is derived from a fundamentally random quantum process. This approach offers an advantage in higher precision, better randomness of the output and conceptual simplicity of circuits.

## Introduction

Today, computing is almost exclusively done via the Digital Computation paradigm (DC), based on Turing machine theoretical model. Implemented in electronics logic circuitry, which executes Boolean logic operations, and realized in solid-state chips, this kind of model allows for a very fast computation with an arbitrary precision. Since DC is incapable of generating randomness, a version enriched by a (single) random number generator, the so-called "randomized Turing machine", offers execution and speedup of certain tasks by using randomized algorithms, for example testing the primality of (large) numbers by Soloway-Strassen algorithm^1,2^.

A radically new, Quantum Computation (QC) paradigm has been proposed by Feynman in 1981^3^. It makes use of strong correlations of quantum entanglement and superposition principle to reach an exponential speed-up over DC of a small but evergrowing set of algorithms of a great practical importance^4^. Input and output information to a QC is digital, however, internally it manages an analog construct: a multi-particle quantum state. The initial quantum state (the problem) is evolved by a set of operations to a final state (the solution), then measured to obtain a statistical output. A large effort is being put on building a universal programmable quantum computer of a precision that would have a practical significance, but thus far technological difficulties have kept that goal out of reach.

A Random Pulse Computing (RPC) paradigm, proposed in a seminal work of John von Neumann^5^ in 1956, makes use of counting pulses that appear randomly in time, thus being similar to nerve pulses of living beings. RPC flashed in 1960s only to be run over by the digital computation that flourished in 1970s. Reborn in mid 2010s^6^, RPC can be thought of as a third computational paradigm, alongside to DC and QC paradigms. Variations of the initial RPC led to development of a large set of techniques known as *stochastic computing*^7–9^.

The main drive behind the recent revival of the RPC is a hope that it could efficiently (in terms of execution time, amount of hardware and energy consumption) solve problems that seem difficult and/or energy-consuming for the DC, but apparently easy for living beings, such as: deep learning that mimic human brain operation^10^, digital signal processing filters^11^, edge detection and noise reduction in visual images ^12^, fault-tolerant computation^13^, hybrid neural network for sensor data processing, using near-sensor stochastic computation for data reduction, followed by precision binary computation^14^, as well as for energy and space efficient, fault-immune neural networks for general tasks^15^. However, unlike for DC circuits, a general theory of synthesis and analysis of RPC circuits is still missing. Even though attempts have been made towards general functional synthesis via approximate decomposition to Bernstein polynomials^16^ or via a spectral transform approach^17^, these approaches cannot be automated since they require guesswork and/or ad-hoc optimizations. As a thrilling complication, it has been noted that the spectral transform approach may end up in two or more different Boolean circuits (which have different truth tables) that realize the exact same RPC function^17^.

**Figure 1:**
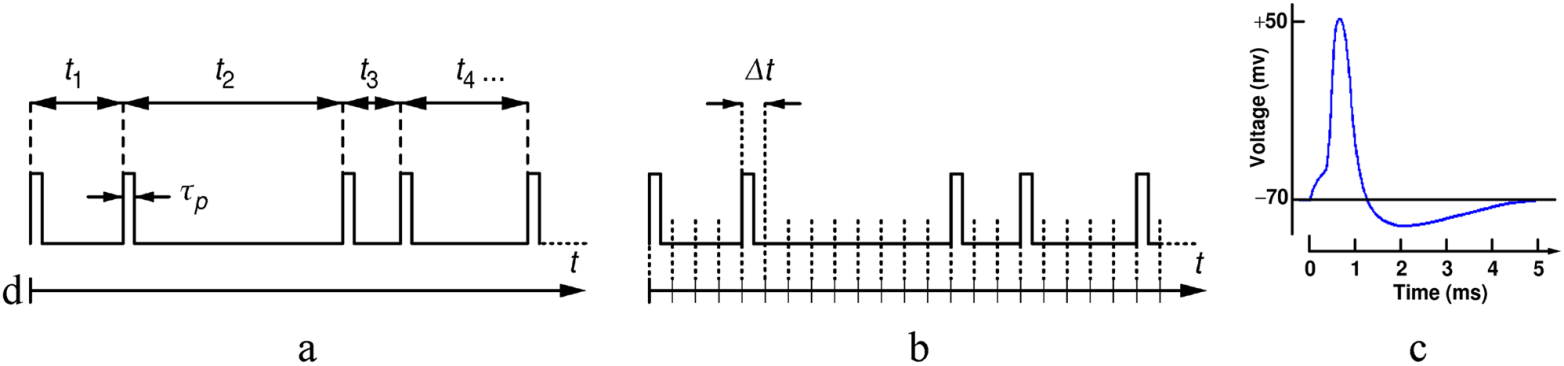
Random pulse train in which each pulse corresponds to a Poissonian random event with frequency $1/\langle {t}_{i}\rangle$ **(a)**; time-discrete pulse train wherein appearance of a pulse in a given time segment of width $\Delta t$ is an independent binomial random event with probability $p$ **(b)**; one full cycle of a biologic nerve pulse in mammalians that lasts about 5 ms **(c)**.

In the RPC, any input, output or intermittent numerical value is encoded in a random pulse train (RPT). In its original appearance^7,18^, the RPT was a train of square electric pulses of a fixed height and width that appear randomly in time. In this work we use the so-called "unipolar" representation of the random pulse train whose pulse shape most closely resembles pulses found in synapses of mammalian nerve cells. Other representations known in literature are bipolar and stochastic single-line representations^7^, as illustrated in Fig. 1a. Each pulse is generated upon a Poissonian random event. Such events can be obtained from pseudo-random sources^19^, certain types of electronics noise^8^, but for the best randomness they should preferably be derived from a discrete quantum-random process such as decay of a nuclei^20^, single-photon detection^21^ etc. The only parameter of such a pulse train (PT) is its pulse rate, which represents a number in the RPC computer.

The RPC computer performs calculation through a series of interconnected basic operation circuits which can be conveniently realized by Boolean logic circuits (OR, AND, NOT…). However, logic operations performed on digital pulses that can appear at any time, will generally result in pulses of variable duration or glitches which are unfit for further use, which represents a great technological inconvenience. The problem can be eliminated by discretizing the timeline into segments of duration $\Delta t$, as shown in Fig. 1b, and assigning a pulse to each time segment through a binomial process with a constant probability $p$. This ensures that pulses from various pulse trains appear neatly aligned in time so that they can be reliably processed by logic circuits. Such pulses show striking resemblance to electrical nerve pulses shown in Fig. 1c. The "clock", of frequency ${f}_{\mathrm{CLK}}=1/\Delta t$, is now defining the time.

In this work we differ random pulse trains generated by the binomial process (RPT) and general pulse trains (PT) generated by any stationary process. The numerical information carried by a PT is its pulse probability $p\in \left[\mathrm{0,1}\right]$. If a different numerical range is required for an application, some sort of mapping must be applied.

Even though RPC computer uses digital pulses and randomness, just as probabilistic DC does, it radically differs from it. Firstly, with a difference that information is not in the form of a logic state of a register, but in the form of the RPT, the RPC fundamentally uses the time as a new dimension in calculation. Secondly, there is an enormous difference in amount of hardware required to perform mathematical operations. For example, simple ANDing of two time-discrete RPTs results in multiplication of two real numbers (as will be explained in “Relative KS entropy”), while multiplication of two floating-point numbers in DC would require a circuit made of hundreds of gates.

Thus far, precision of the basic RPC circuits has been analyzed assuming RPTs^7–9,18,22^ or assuming specifically tailored strongly correlated PTs^16^. In doing so, it has been noted that some RPC circuits generate correlated (non-random) output PTs and if such PTs are fed as input to further RPC circuits, a large computation error may occur^9,22,23^. To tackle the problem of calculation accuracy, for the first time to the best of our knowledge, we investigate the operation of RPC circuits from a perspective of their input and output entropies, introducing notions of *relative KS entropy* and *entropy budget*, defined below. We also find a criterion for existence of a RPC circuit for a given mathematical operation.

## Experimental setup and methods

The specific difference of our approach to the research of RPC circuits, with respect to the state of the art, is that we use quantum randomness for generation of RPTs. The source of randomness is a photoelectric effect^24^ in which light of constant macroscopic intensity falls upon a single-photon avalanche photodiode (SPAD) causing a stationary detection process. While the average rate of a photon detection is proportional to the intensity of light, as explained in Ref.^24^, there is fundamentally no parameter of the system that could possibly give any information about *when* a photon is to be detected. Since the only parameter that describes the detection process is its rate, it is a Poissonian process, ideally suited for creation of an RPT described above.

**Figure 2:**
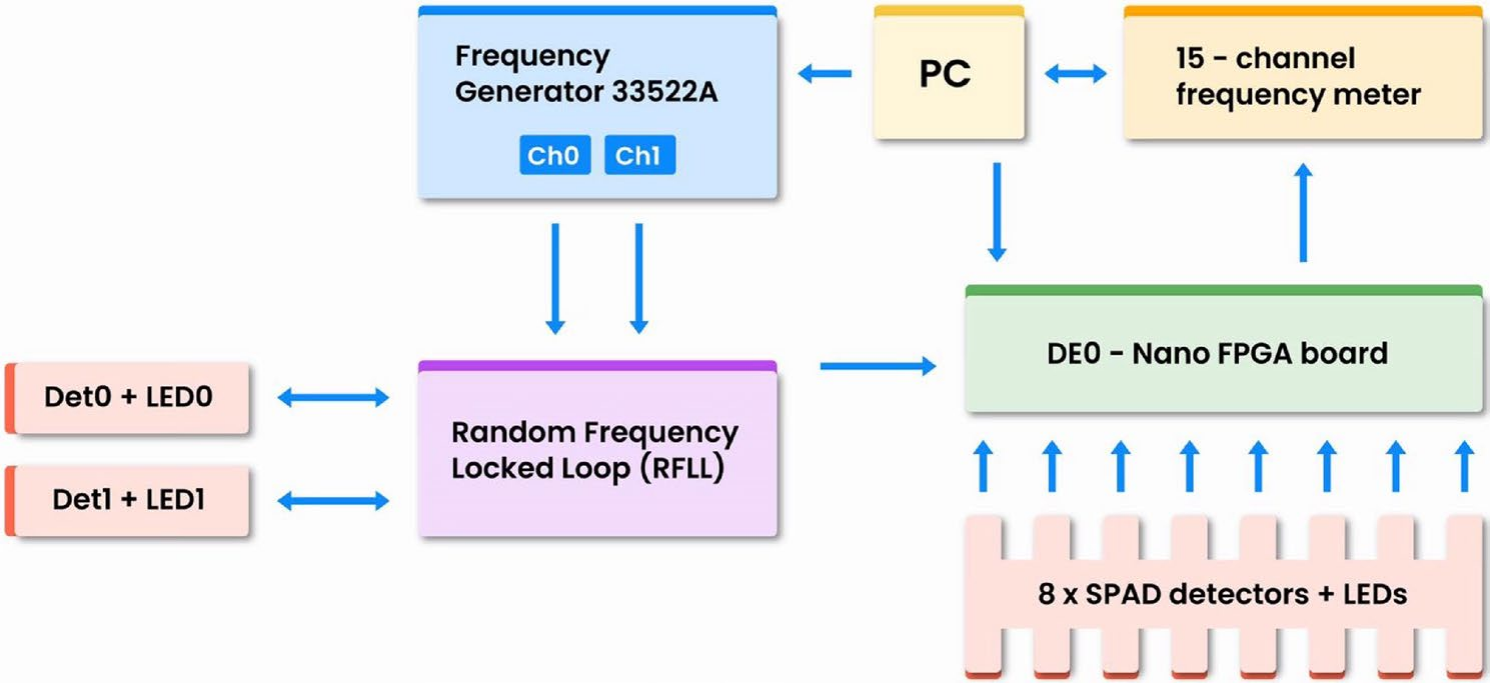
Setup for experimental implementation and testing of RPC circuits.

The heart of the setup, shown in Fig. 2, is the DE0-Nano board (Terasic) containing the Intel-Altera Field Programmable Gate Array (FPGA) chip of the *Cyclone IV* family with $\mathrm{22,320}$ macro cells and four Phase-locked loops (PLLs) each of which can generate up to 3 synchronized clock signals with arbitrary mutual phases. The RPC circuits are realized within this programmable chip.

The main system clock (CLK), that defines time discretization, has a duty cycle of 50% (pulse duration of 500 ns). It is generated by a PLL, available within the FPGA. We have chosen the rate of ${f}_{\mathrm{CLK}}=1$ MHz for the clock, thus time segments, shown in Fig. 1b, have duration of $\Delta t=1 \mathrm{\mu s}$.

To begin with, we use light-emitting diode (LED) for the light source from Hamamatsu (model L7868, peak wavelength ${\lambda }_{0}=670$ nm, FWHM spectral width $\Delta \lambda =30$ nm), operated in continuous wave (CW) mode of constant intensity. For our purpose, the most important feature of the light source is that the photon emission times are not mutually correlated and therefore can be considered random. For the light source with a Gaussian emission spectrum, the coherence time is given by^25^:

1 $${\tau }_{c}=\frac{1}{c}\frac{{\lambda }_{0}^{2}}{\Delta \lambda }$$

and for our source, it amounts about $50$ fs. This means that detections should be uncorrelated as long as our photon detection rate is significantly lower than $1/{\tau }_{c}\approx 20$ Tcps. In our setup, we use detection rates less or equal to 16 Mcps, thus being over 6 orders of magnitude below the rate at which temporal correlations may appear.

Secondly, in a SPAD, each incoming photon has a well-defined probability (aka. quantum efficiency) of generating one free carrier. The carrier is internally amplified, via the avalanche mechanism, to give a sizeable current signal, which is subsequently amplified and shaped into a digital pulse. We use a virtually afterpulse-free SPAD, model SUR500 from Laser Components, in a home-made single-photon detector (SPD) with active quenching circuit similar to the one described in Ref.^26^. These SPDs provide a clean detection signal with afterpulsing probability $<0.02$%, a typical dark count rate of 16 kcps and dead time of 30 ns. In our setup, random pulses generated by an SPD are used either to generate time-discrete RPT or as an input to the so-called *random flip-flop* (RFF)^27^, a stochastic logic circuit which we use in RPC circuits to add entropy or to make random decisions.

For generating RPTs, we use two SPDs (Det0 and Det1), which are illuminated by two independent LEDs (LED0 and LED1) operated in CW mode with adjustable intensity. Due to the randomness of photon emission from LEDs, detectors generate RPTs of high randomness. The average count frequency of each detector can be controlled by a personal computer (PC) via a frequency generator 33522A (Keysight), in the range from 20 kHz to 5 MHz, using the random-frequency-locked-loop (RFLL) approach^28^. The output signal of each detector is fed into the FPGA and a pulse is generated within a time segment $\Delta {t}_{i}$ if and only if one or more pulses have been received during the previous time segment $\Delta {t}_{i-1}$. This allows us to generate two independent RPTs with probabilities ${p}_{0}$ and ${p}_{1}$ in the range of $0.02$—$0.98$, independently settable by the PC. We use those two RPTs as input variables to the tested RPC circuits.

**Figure 3:**
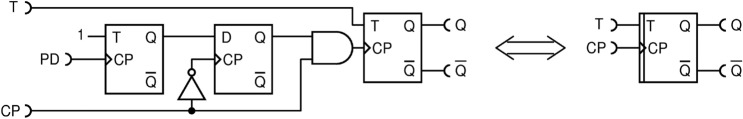
Realization of the T-type random flip-flop (TRFF): T and CP are its inputs, $\mathrm{Q}$ and $\overline{\mathrm{Q} }$ are its outputs, while PD is an "internal" input (not shown in the symbol) that receives about 16 Mcps random pulses from a photon detector, situated outside of the FPGA and illuminated by a constant intensity light from an LED. Symbol of a T-type RFF is shown on the right end.

A further eight SPDs, which add to total of ten SPDs included in the setup, are used to realize eight independent T-type random flip flop (TRFF) circuits whose schematic is shown in Fig. 3. Each SPD is illuminated by its own LED in the constant-intensity mode, so that it detects photons at a fixed rate ${f}_{\mathrm{PD}}\approx 16$ Mcps. The photon detection rate ${f}_{\mathrm{PD}}$, being much higher than ${f}_{\mathrm{CLK}}$, ensures random operation of TRFFs^29^.

Finally, outputs from the FPGA are being fed to a home-made 15-channel frequency meter connected to the PC, thus up to 15 different RPC circuits can be simultaneously measured and their output probabilities recorded. The multi-channel operation enables to cut down acquisition times, thus making possible to record detailed transfer functions. Since this system is not sensitive to distribution of pulses on a timeline, an additional time-tagger ID900 from IdQuantique (not shown in the setup) was used to reconstruct times at which individual pulses arrive in a pulse train and store this information to the PC. This information is used for estimation of output entropies of various RPC circuits, as detailed in the next section.

We also wrote from scratch a computer program for simulation of RPC circuits using Monte Carlo method. i.e. method of repeated random sampling. It enabled us to evaluate and debug a large ensemble of circuit candidates before embarking on a time-consuming practical realization and experimental testing of those that have shown satisfying performance.

## Entropy considerations

A time-discrete PT generated has a well-defined pulse probability. Over time an $N$-dimensional vector of pulses/bits ${\varvec{x}}=({x}_{0},\boldsymbol{ }{x}_{1},\dots ,\boldsymbol{ }{x}_{N-1})$ is created which fully represents the PT in the following manner: ${x}_{i}=1$ signifies a pulse in the $i$-th time bin, while ${x}_{i}=0$ signifies no pulse. Shannon entropy of such a bitstream, seen as a sequence of 1-bit symbols is given by:

2 $${H}_{1}\left({\varvec{x}}\right)=\mathcal{H}\left(p\left({\varvec{x}}\right)\right):=-p({\varvec{x}}){\mathrm{log}}_{2}p({\varvec{x}})-\left(1-p({\varvec{x}})\right){\mathrm{log}}_{2}\left(1-p({\varvec{x}})\right) ,$$

where the pulse probability is

3 $$p\left({\varvec{x}}\right)=\frac{1}{N}\sum_{i=0}^{N-1}{x}_{i} . $$

This definition of entropy, which considers words of a length equal to $1$ bit, is satisfactory, namely a good measure of randomness of the bitstream, in case when bits are generated in a statistically independent manner, such as in an RPT. In that case, entropy of 1 means maximally random binary sequence ${\varvec{x}}$ (achieved for $p=1/2$), a lesser value of entropy means a lesser randomness, and zero entropy means complete absence of randomness, i.e., a deterministic sequence (achieved for $p=0$ or $p=1$). However, in a general case of a pulse train, entropy of a binary string can only be properly grasped by considering longer words. For example, infinite-length toggling train 0101010101…, following Eq. (2), suggests entropy of 1. However, when looked like a sequence of 2-bit words, it is just a repetition of word "01" thus making it apparent that this sequence contains no entropy, and therefore for a general case we need a different definition of entropy. Shannon entropy of $n$-bit words, in literature, also known as "$n$-grams", is defined as

4 $${H}_{n}\left({\varvec{x}}\right)=-\sum_{i=0}^{{2}^{n}-1}{p}_{i}{\mathrm{log}}_{2}{p}_{i} ,$$

where ${p}_{i}$ is a probability of finding $i$-th of the possible ${2}^{n}$ $n$-grams starting at any position in ${\varvec{x}}$**.** Following Ref.^30^, we introduce conditional entropies as the average information necessary to predict the next *n*-gram given the whole bitstream ${\varvec{x}}$

5 $${h}_{n}\left({\varvec{x}}\right)={H}_{n}\left({\varvec{x}}\right)-{H}_{n-1}\left({\varvec{x}}\right) ,$$

for $n\ge 2$ and ${h}_{1}\left({\varvec{x}}\right)\equiv {H}_{1}\left({\varvec{x}}\right)$, where it is also assumed that the length of ${\varvec{x}}$ goes to infinity. This definition implies that conditional entropies form a monotonic non-increasing sequence^30^

6 $${h}_{n+1}\left({\varvec{x}}\right)\le {h}_{n}\left({\varvec{x}}\right). $$

Of particular interest is the entropy of the source, also known as Kolmogorov-Sinai entropy (henceforth *KS entropy*, or just *entropy* where not ambiguous), defined as

7 $$h\left({\varvec{x}}\right):=\underset{n\to \infty }{\mathrm{lim}}{h}_{n}\left({\varvec{x}}\right) .$$

The interpretation of $h$ is the average information necessary to predict the next bit, given all information about the dynamical system that generates the sequence of number ${\varvec{x}}$^30,31^. Because of monotonicity in Eq. (6), most of ${h}_{n}$ are very close or equal to $h$ for large enough *n*. Therefore, $h$ can be expressed as average ${h}_{n}$ in the limit of $n\to \infty$

8 $$h\left({\varvec{x}}\right)=\underset{n\to \infty }{\mathrm{lim}}\frac{1}{n}\sum_{j=1}^{n}{h}_{j}\left({\varvec{x}}\right)=\underset{n\to \infty }{\mathrm{lim}}\frac{{H}_{n}\left({\varvec{x}}\right)}{n} .$$

The last term, obtained by evaluation of the sum through iterative application of Eq. (5), tells us that the KS entropy is equal to the normalized Shannon entropy in the limit of infinitely large $n$-grams. In order to determine KS entropy of PTs obtained experimentally or by simulations, we wrote a computer program that calculates entropy according to Eq. (8).

It is important to note that for a system which contains a finite quantity of information, say $M$ bits, conditional entropies ${h}_{n}$ eventually fall to zero, thus $h=0$. All deterministic systems fall into this category, including pseudo-random ones. Only a system that contains an inexhaustible source of information can have $h>0$. An example of such a system is the TRFF circuit, shown in Fig. 3, which employs quantum randomness.

### Relative KS entropy

From all discrete pulse trains ${\varvec{x}}$ with pulse probability $p({\varvec{x}})$, binomial RPT defined as in Fig. 1b, has the maximal KS entropy. In practice, RPC circuits may produce PTs whose KS entropy is not maximal. For purpose of evaluating and comparing performance of a RPC circuits, we define *relative KS entropy* of an arbitrary PT ${\varvec{x}}$ as

8a $${h}_{rel}\left({\varvec{x}}\right)=\frac{h\left({\varvec{x}}\right)}{{h}_{1}\left({\varvec{x}}\right)} . $$

By virtue of Eq. (6), one finds that ${h}_{rel}$ lies within interval [0, 1]. It gives us a measure of how high entropy of a pulse train is with respect to the maximum entropy it could have with a given pulse probability. The maximum value of ${h}_{0}\left({\varvec{x}}\right)=1$ is achieved when pulses in ${\varvec{x}}$ are generated by a binomial process in which case $h\left({\varvec{x}}\right)={h}_{1}\left({\varvec{x}}\right)={H}_{1}\left({\varvec{x}}\right)=\mathcal{H}(p({\varvec{x}}))$, while value of 0 indicates deterministic generation process.

### Entropy budget criterion

**Figure 4:**
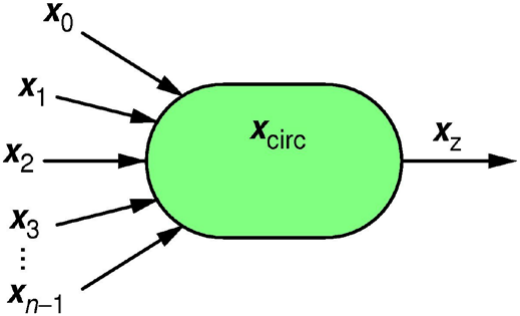
Possible entropy sources in an RPC circuit, available for generation of the output RPT ${{\varvec{x}}}_{z}$, are the input RPTs ${{\varvec{x}}}_{0,}\dots ,{{\varvec{x}}}_{n-1}$, and an internal entropy source(s) ${{\varvec{x}}}_{\mathrm{circ}}$.

Central to this study of RPC circuits, we introduce a concept of *entropy budget*. Firstly, let us consider an $n$-input RPC circuit with input RPTs ${{\varvec{x}}}_{0},\dots ,{{\varvec{x}}}_{n-1}$ and an internal RPT ${{\varvec{x}}}_{\mathrm{circ}}$, as shown in Fig. 4. For simplicity, but without loss of generalization, we consider only one internal RPT.

The *Independence entropy bound theorem* or IEBT (see e.g. Equation (2.96) in^32^) states that the total available entropy is less than or equal to the sum of input entropies:

9 $$h\left({{\varvec{x}}}_{0},\dots ,{{\varvec{x}}}_{n-1},{{\varvec{x}}}_{\mathrm{circ}} \right)\le h\left({{\varvec{x}}}_{\mathrm{circ}}\right)+\sum_{i=0}^{n-1}h\left({{\varvec{x}}}_{i}\right),$$

with equality sign holding true in the case when all entropy sources are statistically independent of each other. We note that even though IEBT in^22^ is proven for Shannon entropy, it holds for KS entropy as well, because of the second equality in Eq. (8). Therefore, entropy of output ${{\varvec{x}}}_{z}$ is less than or equal to the total available entropy:

10 $$h\left({{\varvec{x}}}_{z}\right)\le h\left({{\varvec{x}}}_{\mathrm{circ}}\right)+\sum_{i=0}^{n-1}h\left({{\varvec{x}}}_{i}\right) .$$

Equation (10) holds true for any RPC circuit. Secondly, let us now consider an arbitrary multivariable function ${p}_{z}({\varvec{p}})$, where ${\varvec{p}}={(p}_{0},\dots ,{p}_{n-1})$ is an $n$-dimensional vector of pulse probabilities associated with input RPTs ${{\varvec{x}}}_{0,}\dots ,{{\varvec{x}}}_{n-1}$, and ask whether a circuit that would output an RPT ${{\varvec{x}}}_{z}$ having a pulse probability $p({{\varvec{x}}}_{z})$ can exist. According to Eq. (10) and noting that for an RPT $h\left({\varvec{x}}\right)={H}_{1}\left({\varvec{x}}\right)=\mathcal{H}(p({{\varvec{x}}}_{z}))$, for any such circuit it must hold:

11 $$\mathcal{H}(p({{\varvec{x}}}_{z}))\le h\left({{\varvec{x}}}_{\mathrm{circ}}\right)+\sum_{i=0}^{n-1}h\left({{\varvec{x}}}_{i}\right). $$

Finally, we name Eq. (11) the *entropy budget criterion* (EBC): if it is not fulfilled, no circuit may exist that would perform function ${p}_{z}({\varvec{p}})$. Note that for a deterministic RPC circuit, by definition, the internal entropy $h\left({{\varvec{x}}}_{\mathrm{circ}}\right)=0$.

### Computation errors: systemic and statistical

There are two mutually independent sources of computation errors in an RPC circuit. The statistical error comes from the fact that the output RPT is a binomial variate measured or available over a finite time, say $N$ clock intervals, and thus the variance of the output probability ${p}_{z}$ is given by ${\sigma }^{2}\left({p}_{z}\right)=N{p}_{z}(1-{p}_{z})$. The systemic error is caused if circuit calculates the desired function in an approximate way. In this work, we are solely interested in the systemic error: the one that is intrinsic to the circuit and persists even when $N$ tends to infinity.

### Dynamic optimality

We say that a mathematical operation ${p}_{z}({\varvec{p}})$ is *dynamically optimal* if for the whole set of allowed input values ${\varvec{p}}$(the *domain*), the set of possible output values (the *image*) satisfies two conditions: (a) it is a subset of interval $\left[0, 1\right]$; (b) it includes 0 and 1. For example, multiplication of $n$ numbers ${p}_{i}\in \left[\mathrm{0,1}\right]$, where $i=0,\dots n-1$, is dynamically optimal: it reaches minimum of 0 if at least one number is 0, while it reaches maximum of 1 when all numbers are equal to 1. Dynamic optimality is an important consideration in circuit design, whose purpose is to minimize computation errors.

## Results

Basic arithmetic RPC circuits perform elementary binary operations: addition, subtraction, multiplication and division, and are a necessity towards building a universal RPC-based computer. While addition and multiplication can be performed without approximation and with relatively simple circuits, division and subtraction use approximate approaches, which result in erroneous calculation. The question is whether the precision can be improved, and at which cost. To preserve advantages of the RPC, in the design of novel circuits, one should take care of the following requirements:

1. Minimize computation error over the whole state space of input parameters;
2. Minimize deviation from the Binomial process at the output;
3. Minimize quantity of hardware required to build the circuit.

Of course, these three requirements are generally pairwise exclusive, thus generating a Mexican standoff situation. Therefore, generating new and/or improved circuits for the RPC is not trivial. Here, we present circuits for mathematical operations as well as their entropy budgets starting from multiplication and addition, whose circuits are already well known, to division and subtraction, where we introduce novel circuits, along with the magnitude comparator.

Additionally, the simplest unary operation is negation: in an RPT with pulse probability $p,$ it replaces each pulse with no-pulse and each no-pulse with a pulse, effectively calculating operation $1-p$. It is performed by the NOT logic circuit, as will be shown in Sect. 3.4.

### Multiplication circuits

**Figure 5:**
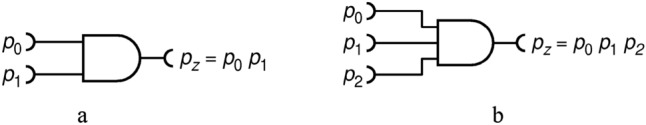
Circuits for exact multiplication of two numbers **(a)**; and three numbers **(b)**.

Multiplication is the simplest operation in the RPC. It is well known that it can be performed with an AND gate, as shown in Fig. 5a ^18^. If pulses with probabilities ${p}_{0}$ and ${p}_{1}$ from RPTs are independent, a probability to have two such pulses in the same time segment is just ${p}_{0 }\cdot {p}_{1}$. Therefore, this circuit gives an exact result, and its output is an RPT. Moreover, this circuit can be upgraded to multiply more numbers simultaneously (not in a cascade) by adding the required number of inputs to the AND gate, as shown in Fig. 5b. This circuit is dynamically optimal: it reaches 0 if any input is equal to 0, while it reaches 1 only if all inputs are equal to 1.

Multiplication circuits of Fig. 5 satisfy the EBC. This is proven in the Appendix I for multiplication of two variables. By cascading that result, it is easy to see that the EBC holds for any number of variables, that is

12 $$hp\left({{\varvec{x}}}_{0}\&{{\varvec{x}}}_{1}\&\dots \&{{\varvec{x}}}_{n-1}\right)\le \sum_{i=0}^{n-1}h\left({p}_{i}\right) ,$$

where we denote bitwise AND operation with symbol & and ${p}_{z}={p}_{0}\cdot {p}_{1}\cdot \dots \cdot {p}_{n-1}$.

### Addition circuits

The second operation presented in this article is addition. Sum of two probabilities spans the whole interval $\left[0, 2\right]$, while output of an RPC circuit, being itself a probability, cannot surpass 1. Therefore, plain addition is not realizable. Here we discuss two approaches.

**Figure 6:**
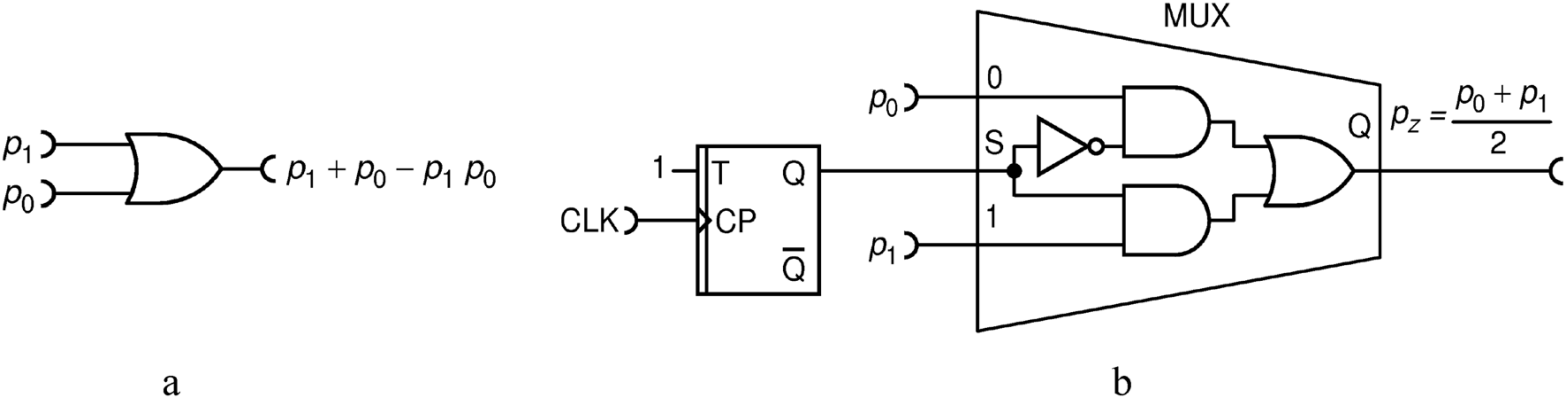
Addition circuits: approximate one with OR gate **(a)**; exact one with a multiplexer (MUX) circuit **(b)**.

The first approach is approximate addition, shown in Fig. 6a. First of all, the OR gate conveys pulses from either input to the output and thus "sums" the two RPTs. But, whenever two pulses from the inputs coincide in time, only one pulse will be formed at the output instead of two. The basic probability calculus yields the expression for the output pulse probability ${p}_{z}$:

13 $${p}_{z}={p}_{0}+{p}_{1}-{p}_{0}{p}_{1} .$$

While this particular binary operation may be useful in its own right, it can also be used to perform an approximate addition since, for sufficiently small ${p}_{0}$ and/or ${p}_{1}$, the multiplicative term becomes negligible. Furthermore, this circuit is dynamically optimal, namely ${p}_{z}\in \left[\mathrm{0,1}\right]$, which can be concluded from the following equality:

14 $${p}_{z}=1-\left(1-{p}_{0}\right)\left(1-{p}_{1}\right) .$$

Due to the statistical independence of the input RPTs, output is also an RPT. Therefore, the output has a maximal relative KS entropy, from which we conclude that the EBC is satisfied.

Lastly, this circuit can be generalized to perform a simultaneous operation on three or more numbers by just adding physical inputs to the OR gate. For example, for three RPTs with probabilities ${p}_{0},{p}_{1},{p}_{2}$ a 3-input OR gate would calculate:

15 $${p}_{z}={p}_{0}+{p}_{1}+{p}_{1}-{p}_{0}{p}_{1}-{p}_{1}{p}_{2}-{p}_{2}{p}_{0}+{p}_{0}{p}_{1}{p}_{2}=1-\left(1-{p}_{0}\right)\left(1-{p}_{1}\right)\left(1-{p}_{2}\right) ,$$

which is again dynamically optimal, generates an RPT and calculates approximate summation in the limit of vanishing input probabilities.

The second approach is fractional summation based on a multiplexer circuit (MUX), as shown in Figure 6b. MUX circuit is well known in the art: it permits to select one of its inputs and forward it to the single output. The selection is done according to the numerical code at the "select" input(s). In our case, we use MUX with two inputs wherein selection is controlled by a random bit. Upon each clock, MUX selects randomly, and with equal probability, the input from which it conveys signal to the output, resulting in the following operation:

16 $${p}_{z}=\frac{{p}_{0}+{p}_{1}}{2} . $$

Apart from implementation imperfections, this circuit performs half-sum exactly. It is also dynamically optimal. Randomness of the selection process is crucial for the output to be an RPT. The extra factor 1/2 can be, in principle, counted in a calculation and this should not present a problem. However, a caution needs to be exercised because this kind of binary (2-input) addition is not associative:

17 $$\frac{1}{2}\left(\frac{1}{2}\left({p}_{0}+{p}_{1}\right)+{p}_{2}\right)\ne \frac{1}{2}\left({p}_{0}+\frac{1}{2}\left({p}_{1}+{p}_{2}\right)\right) .$$

**Figure 7:**
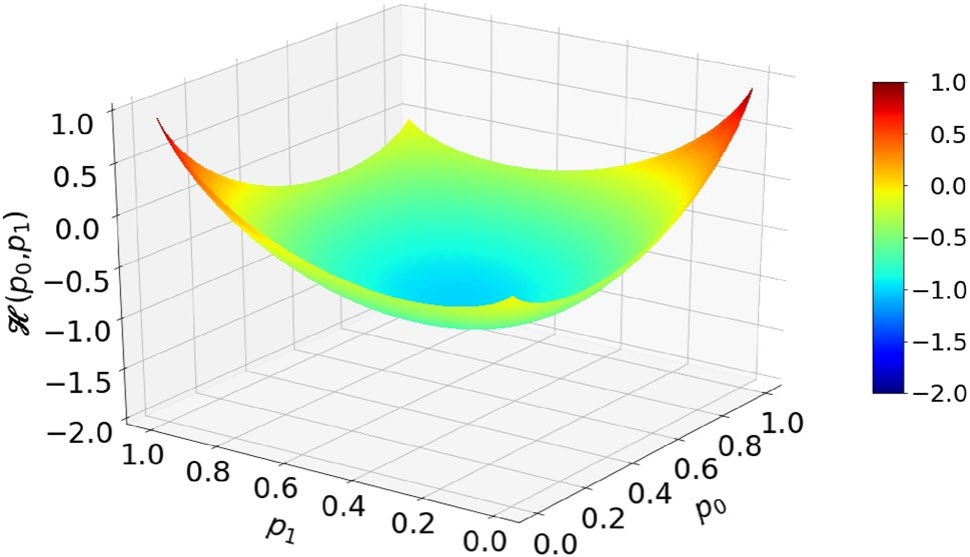
Entropy budget of a summation circuit with inputs ${p}_{0}$ and ${p}_{1}$ that would output ${p}_{z}={(p}_{0}+{p}_{1})/2$. Plot shows the function $\mathcal{H}\left({p}_{0},{p}_{1}\right)=\mathcal{H}({p}_{z})-\left(\mathcal{H}\left({p}_{0}\right)+\mathcal{H}\left({p}_{1}\right)\right)$.

According to the EBC, a deterministic circuit that would perform operation in Eq. (16) is impossible. This is illustrated by theoretical calculation shown in Fig. 7. The orange-red tongues indicate areas of input variables for which the total entropy of inputs is less than the entropy of output. In particular, input combinations ${p}_{0}=0, {p}_{1}=1$ and ${p}_{0}=1, {p}_{1}=0$ have a zero total entropy, while the output entropy should be 1 (per time segment). From that, one concludes that a feasible circuit must contain an internal entropy source capable of generating at least entropy of 1.

On the other hand, if input probabilities are halved, as effectively done by the RFF in the circuit shown in Fig. 6b, the entropy budget inequality becomes satisfied, that is

18 $$\mathcal{H}\left(\frac{{p}_{0}+{p}_{1} }{2}\right)\le \mathcal{H}\left(\frac{{p}_{0}}{2}\right)+\mathcal{H}\left(\frac{{p}_{1}}{2}\right) . $$

Inequality Eq. (18) is strictly proven as Theorem 2 in Appendix II. We note that the clocked RFF circuit acts as an entropy source of exactly 1, so the equality in Eq. (11) may be achieved even in the red parts. This is an example of a circuit that uses internal source of entropy in order to meet the EBC.

One could ask here whether injecting entropy of 1 with each clock is an optimal use of resources. Namely, a deterministic circuit which operates correctly, for the region of input parameters under the green and blue parts of the surface in Fig. 7, is allowed by the EBC. For example, the input parameter point ${p}_{0}=0.45$, ${p}_{1}=0.3$ is well within the green–blue area. The total input entropy is $\mathcal{H}\left(0.45\right)+\mathcal{H}\left(0.3\right)\approx 0.993+0.881=1.874$, while the output entropy is $\mathcal{H}\left(0.375\right)=0.954.$ Thus, for calculation at this point, no additional entropy is needed. The problem is that, unlike in Boolean logic, where a circuit performing any function can be systematically synthesized and minimized^33^, there is no such theory for the randomized Boolean logic yet. Thus, construction of RPC circuits is left to intuition and guesswork.

Finally, in principle, the fractional summation circuit can be generalized to sum more than 2 inputs. Particularly, a binary tree of $n$ MUX circuits can be used to realize a ${2}^{n}$-input summation circuit, so the sum would appear divided by ${2}^{n}$.

### Division circuits

**Figure 8:**
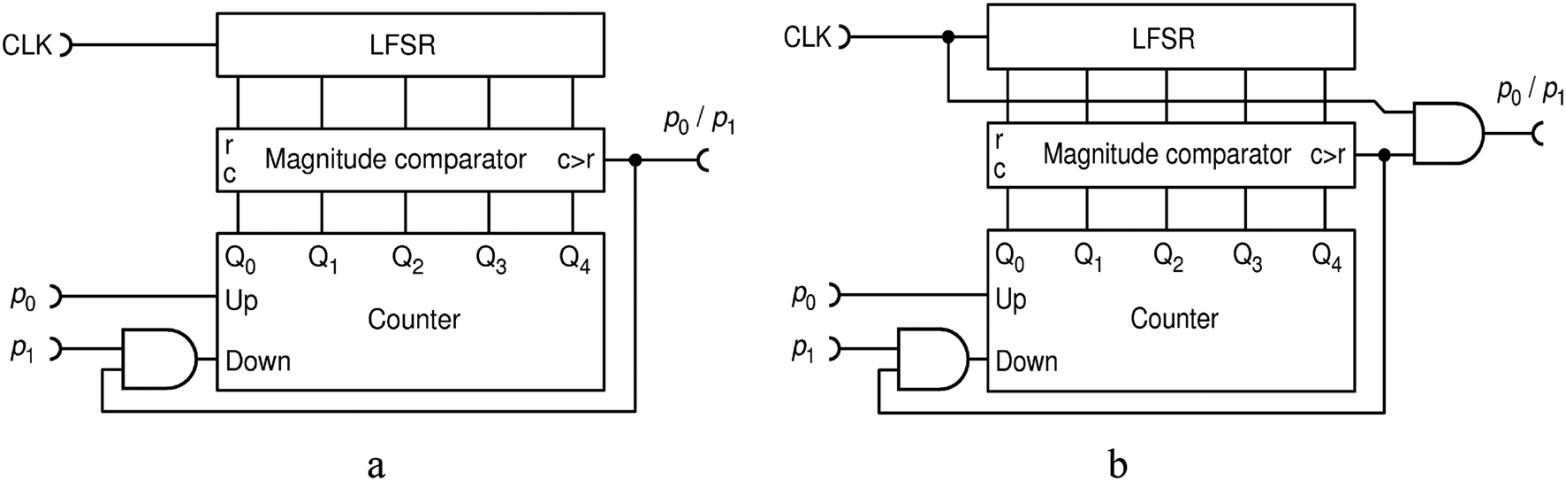
Feedback based division circuit: full-width-pulse version **(a)**; half-width-pulse version (DIV1) **(b)**.

Probably the most important and at the same time most difficult RPC circuit for realization is that of division. Candidate circuits have been extensively studied in the past^22,34^, offering various tradeoffs. For example, one circuit in Ref.^16^ makes use of the *law of total probability* in order to achieve a high precision, but relies on specially prepared, strongly correlated inputs. Here we present three different circuits for division operation which accept uncorrelated RPTs. To begin with, the usual way to realize (approximate) division is through a negative feedback loop, which functions as follows. One starts with a guessed value of ${p}_{z}$ (say 0.5), multiply it with ${p}_{1}$ (we *do* know how to multiply exactly!) and compare the result to ${p}_{0}$. If the result is smaller than ${p}_{0},$ ${p}_{z}$ is enlarged by a small increment, but if it is larger, it is decreased, and the process repeats. This iterative process will lead to an equilibrium around the right solution. A circuit that does that, published in Refs.^7^ and^22^, is shown in Fig. 8a. It consists of three types of complex circuits, well known in standard Boolean logic: an $N$-bit counter, a comparator of two $N$-bit integer numbers (also known as "magnitude comparator") and a Linear Feedback Shift Register (LFSR) that generates a pseudo-random $N$-bit integer number. This particular circuit generates pulses of width $\Delta t$ (instead of $\Delta t/2$), because of which consecutive pulses are "glued" into one long pulse, thus lowering the apparent number of pulses for downstream RPC circuits that use a counter (such as this one). To avoid this pitfall and to keep uniform format of pulses throughout the setup, we use a slightly modified version shown in Fig. 8b., named DIV1, which generates standard pulses of width $\Delta t/2.$ The two versions perform the same.

We emphasize that it is assumed, throughout this work, that an $N$-bit counter can neither count over its maximum of (${2}^{N}-1$) nor below zero. This can be achieved by a simple control circuitry not shown in the schematics.

**Figure 9:**
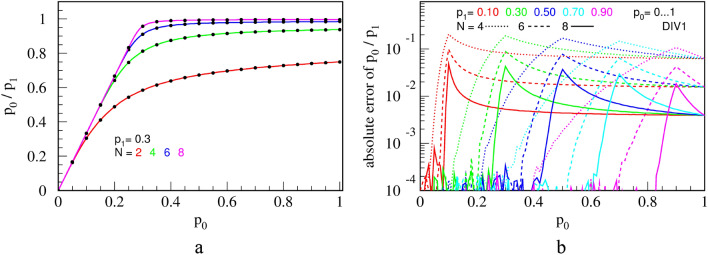
Transfer functions **(a)**; and errors from the ideal division calculation **(b)**, for the circuit DIV1, shown in Fig. 8b. $N$ is the counter capacity in bits.

Moreover, the transfer function and systemic errors of the divider DIV1, obtained experimentally for a certain space of input parameters ${p}_{0}$, ${p}_{1}$ and counter bit of length $N$, are shown in Fig. 9. Here, and in the rest of the presentation, colored curves are obtained by connecting 72 experimentally measured points by a piece-wise straight line. Probability of the output PT is evaluated according to Eq. (3) with statistics of $N=8\cdot {10}^{7}$ bits (time intervals) inf order to reach the statistical error of only $1.1\cdot {10}^{-4}$.

The black dots, shown exclusively in Fig. 9a, represent a Monte Carlo simulation. Measurements and simulation coincide within the statistical error. We made the same check for all subsequent circuits to make sure that both our simulations and practical circuits work correctly.

Ideally, the transfer function should consist of two lines that meet at a sharp knee: ${p}_{z}={p}_{0}/{p}_{1}$ for ${p}_{0}{\le p}_{1}$ and ${p}_{z}=1$ for ${p}_{0}{>p}_{1}$. Circuit DIV1 does that only approximately and approximation improves with a usage of a counter with a larger number of bits $N$. But even with a large enough counter to ensure good precision, DIV1 exhibits several setbacks, some of which are discussed in^35^, such as the following. Firstly, the LFSR shifts by one bit with each CLK pulse so consecutive numbers, to which comparators compare to, are not independent. This tends to correlate output bits and lower the output entropy. One way to improve this could be to shift the LFSR by $N$ bits on each CLK pulse (which is technologically cumbersome and requires a longer LFSR) or to use $N$ independent LFSRs (which is resource-expensive). Secondly, one cannot use the same LFSR for multiple circuits because their outputs would become cross-correlated. Finally, a LFSR must be seeded with a non-zero random number to operate. In order to avoid cross-correlations, seeds should be different for each LFSR or LFSRs should all be different, which would greatly complicate the RPC computer.

**Figure 10:**
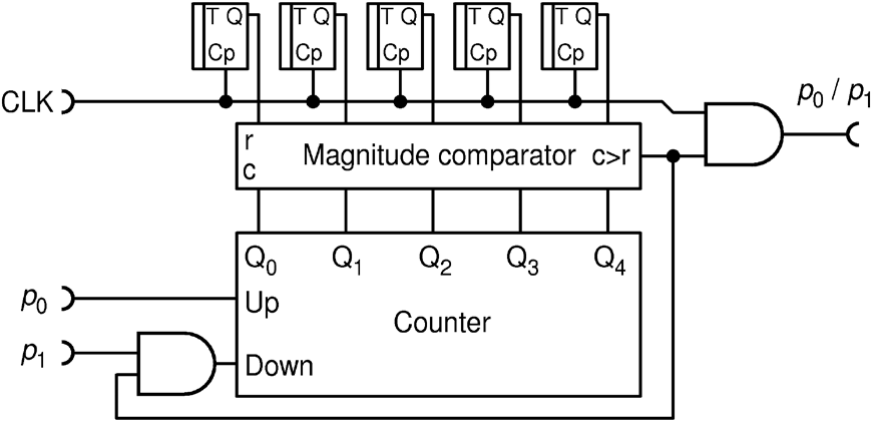
The improved division circuit DIV2 uses of T-type random flip-flops as internal source of entropy.

In order to avoid these pitfalls all in one stroke, here we propose to use true randomness implemented via RFFs, as shown in Fig. 10. According to^27^, a T-type RFF (TRFF) acts as an ordinary TFF with a difference that its clock input (Cp) acts with probability of 0.5, randomly. Thus, if T input is held HIGH, a TRFF generates an independent, truly random bit upon each clock pulse. For the simplicity of drawing, throughout this paper, we will always assume that input T is held HIGH.

**Figure 11:**
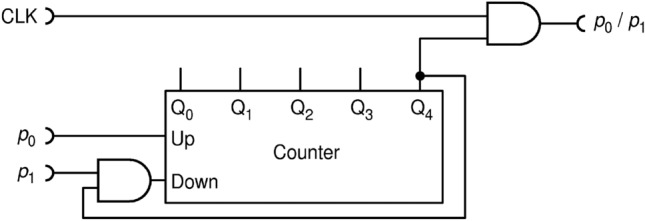
A simple and precise deterministic division circuit DIV3.

Indeed, we find that this circuit, named DIV2, performs better in terms of output randomness than circuit DIV1, but their precision is virtually the same (to within 0.1%) and shown in Fig. 9. To further improve the precision of division and reduce the hardware cost, we propose a new circuit DIV3, shown in Fig. 11.

**Figure 12:**
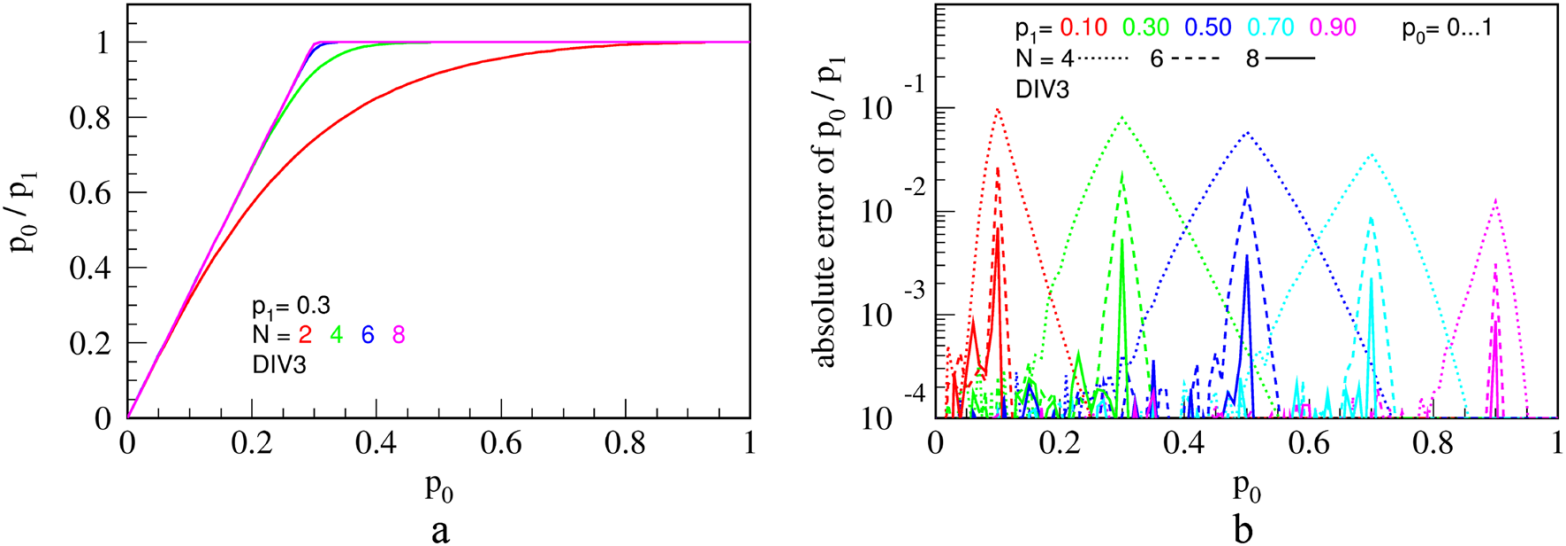
The transfer function **(a)**; and errors from the ideal division calculation **(b)** for the circuit DIV3.$N$ is the counter capacity in bits.

This circuit achieves better precision with a given counter capacity ($N$), while at the same time it does not require sources of randomness nor a resource-expensive digital comparator. Its transfer function and errors are shown in Fig. 12.

Transfer function is significantly improved compared to DIV1 and DIV2 for the same counter capacity $N$ (Fig. 12a). The error, shown in Fig. 12b, is reduced by 1–2 orders of magnitude, peaking only around the "knee", where the circuit counter enters saturation. DIV3 is a deterministic division circuit.

**Figure 13:**
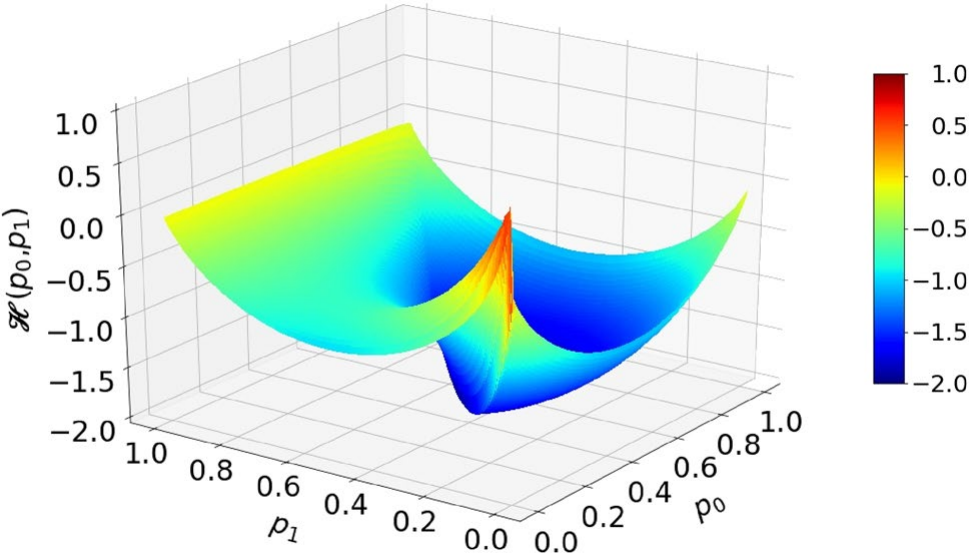
Entropy budget of a division circuit with inputs ${p}_{0}$ and ${p}_{1}$ that would output clipped division ${p}_{z}=\mathrm{Min}\left({p}_{0}/{p}_{1}, 1\right)$. Plot shows the function $\mathcal{H}\left({p}_{0},{p}_{1}\right)=\mathcal{H}({p}_{z})-\left(\mathcal{H}\left({p}_{0}\right)+\mathcal{H}\left({p}_{1}\right)\right)$.

After presenting division circuits, it is important to examine the fulfillment of the EBC. As already mentioned, division is a difficult operation in RPC and only approximate methods to realize it are known. It is so for a couple of reasons. Firstly, division can result in a value larger than 1. Because of that, we will consider a "clipped" division: ${p}_{z}\left({p}_{0}{,p}_{1}\right)=\mathrm{Min}\left({p}_{0}/{p}_{1}, 1\right)$. Secondly, division of two small numbers may yield the output entropy larger than the sum of entropies of the two input numbers, leading to the EBC not being fulfilled. For example, in case ${p}_{0}=0.02, {p}_{1}=0.04\Rightarrow {p}_{z}={p}_{0}/{p}_{1}=0.5,$ we have total input entropy $\mathcal{H}\left({p}_{0}\right)+\mathcal{H}\left({p}_{1}\right)=0.384,$ while output entropy $\mathcal{H}\left({p}_{z}\right)=1$. The EBC for the division is not fulfilled in a sizeable area around point $(0, 0)$, as shown in Fig. 13, which tells us that a deterministic circuit for division is not possible.

**Figure 14:**
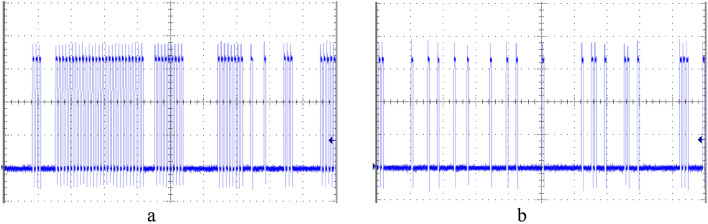
Oscillograms of output RPTs from division circuits DIV3 **(a)** and DIV2 **(b)**, for input values ${p}_{0}=$ 0.3 and ${p}_{1}=$ 0.5.

In this entropy budget analysis, illustrated in Fig. 13, we have proven that a circuit such as DIV3 cannot work correctly. Indeed, since it either passes high frequency pulses from CLK or blocks them, DIV3 tends to generate long bursts of consecutive pulses followed by lengthy periods of absence of pulses, as shown in the oscillogram in Fig. 14a, for the division ${p}_{0}/{p}_{1}=0.3/0.5$. This type of output has a low relative KS entropy. Significantly better randomness is achieved by the circuit DIV2 for the same input variables, as illustrated in Fig. 14b.

**Figure 15:**
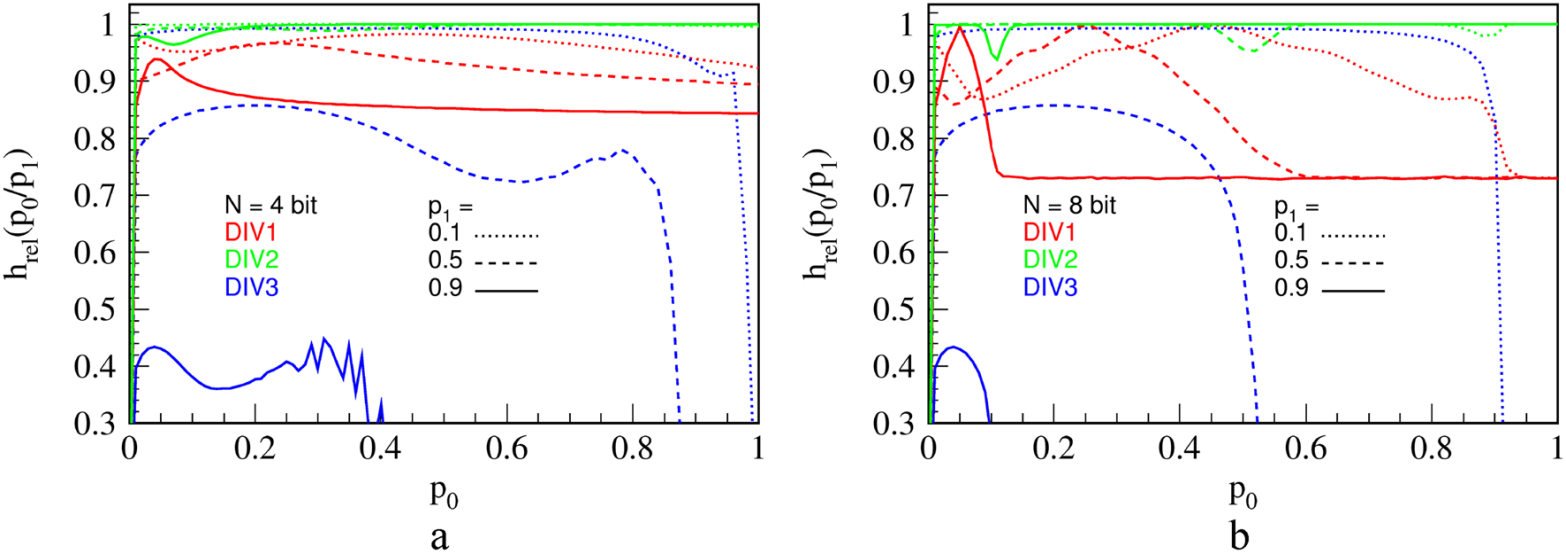
Relative KS entropy of the output of the three division circuits that approximately calculate ${p}_{0}/{p}_{1}$, for ${p}_{1}=$ 0.1, 0.5 and 0.9, for a counter of $N = 4$ bits **(a)** and $N = 8$ bits **(b)**.

To evaluate output randomness from different circuits numerically, we calculate relative KS entropy on long series of RPTs, obtained experimentally from the three division circuits. Results are shown in Fig. 15. For a short counter ($N=4$ bits), we see that DIV2 is by far the best one for virtually any combination of ${p}_{0}$ and ${p}_{1},$ with respect to the output entropy. We note that replacing LFSR in DIV1 with RFFs in DIV2 significantly improves the entropy, and thus randomness, of the circuit. Thus, DIV2 is a clear winner for a general-purpose division. Nevertheless, due to its low hardware cost and high precision, DIV3 is still a valuable circuit. Namely, even though DIV3 generally performs miserably in terms of entropy, it becomes quite good for ${p}_{1}\le 0.1$, where it is roughly equal to DIV1 for a short counter ($N=4$) and even better than DIV1 and close to DIV2 for a long counter ($N=8$). Therefore, if in a complex calculation, input ${p}_{1}$ is limited to about 0.1 for any reason, then DIV3 is an appropriate choice for a divider that saves resources.

### Subtraction circuits

**Figure 16:**
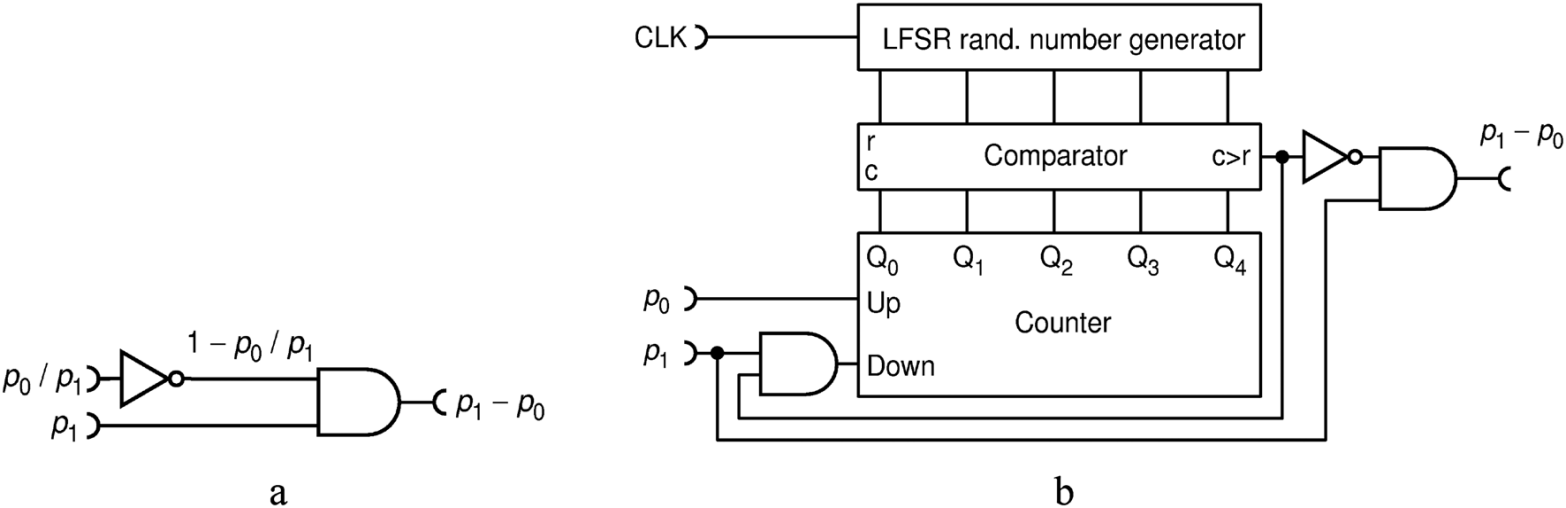
Subtraction via division principle **(a)**; a subtraction circuit SUB1 that uses DIV1 **(b)**.

Subtraction seems to be the most complicated of all basic arithmetic functions in the RPC paradigm because the only known way to perform subtraction is by using the following identity:

19 $${p}_{1}-{p}_{0}=\left(1-\frac{{p}_{0}}{{p}_{1}}\right){p}_{1} ,$$

which includes division. It is important to note that subtraction can result in a value less than zero. Because of that, we will use a "floored" subtraction: ${p}_{z}=\mathrm{Max}\left({p}_{1}-{p}_{0}, 0\right)$. Here we describe three different circuits for subtraction. To begin with, circuit that executes Eq. (19) exactly is shown in Fig. 16a. By inserting the practical division circuit DIV1 shown in Fig. 8b, one arrives to an approximate subtraction circuit shown in Fig. 16b, that we name SUB1.

**Figure 17:**
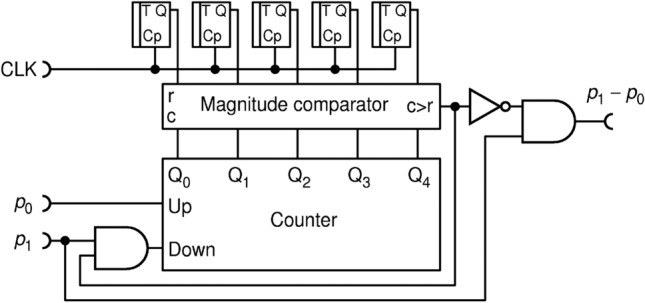
Subtraction circuit SUB2 with an improved output randomness.

Again, improvement in randomness and hardware reduction, without a gain in precision, can be obtained by substituting LFSR with TRFF, as shown in Fig. 17, and we name that circuit SUB2.

**Figure 18:**
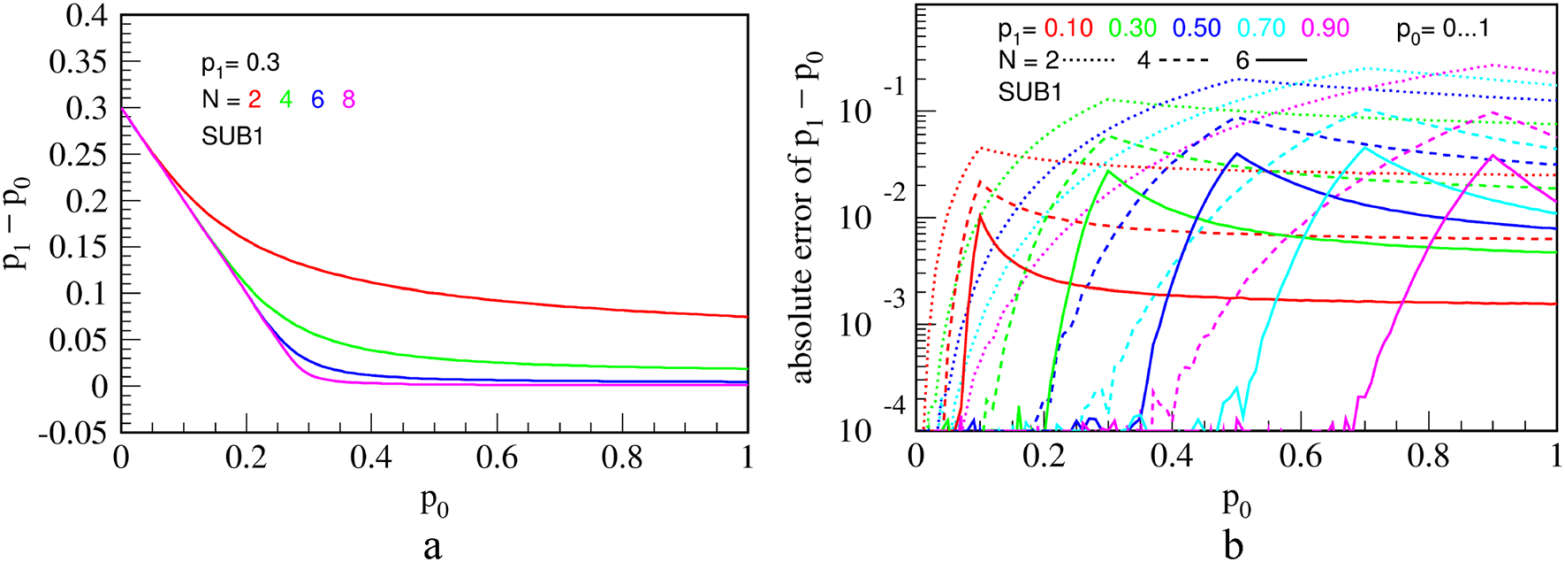
Measured transfer function **(a)**; and errors (difference from the ideal division) **(b)** for the circuit SUB1, for a fixed value of ${p}_{1}=0.3$ and counter capacities of $N=2, \mathrm{4,6}$ and $8$ bits. The results are equal for the improved circuit SUB2.

Transfer function and errors of circuit SUB1, virtually equal to those of SUB2, are shown in Fig. 18.

**Figure 19:**
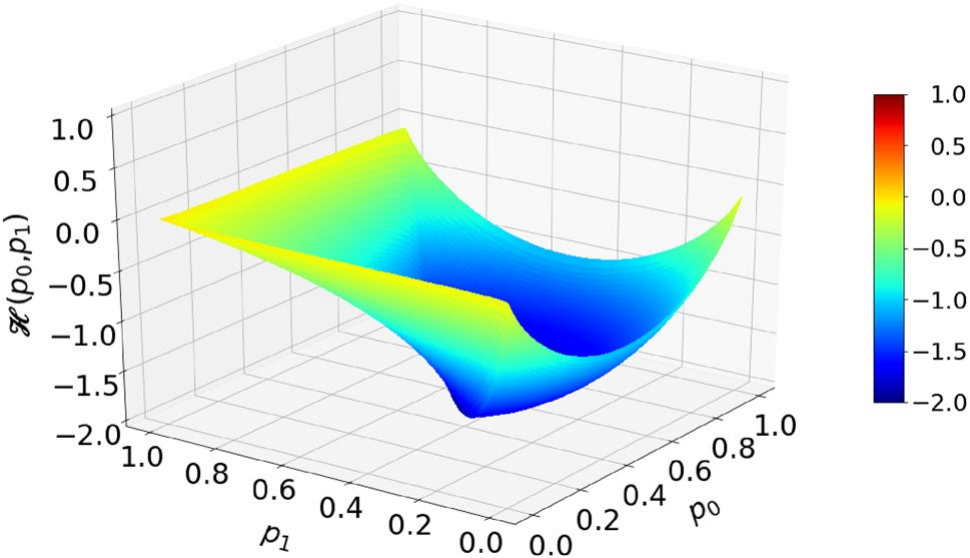
A budget of a circuit with inputs ${p}_{0}$ and ${p}_{1}$ that would output clipped subtraction ${p}_{z}=\mathrm{Max}\left({p}_{1}-{p}_{0}, 0\right)$. Plot shows the function $\mathcal{H}\left({p}_{0},{p}_{1}\right)=\mathcal{H}({p}_{z})-\left(\mathcal{H}\left({p}_{0}\right)+\mathcal{H}\left({p}_{1}\right)\right)$.

Following the fact that deterministic circuit for division is not possible and that a subtraction circuit can be obtained from division and a few deterministic circuits, one might be tempted to conclude that deterministic subtraction circuit is not possible. Surprisingly, numerical analysis of the entropy budget, shown in Fig. 19, reveals that the EBC holds for subtraction, which is proven as Theorem 3 in Appendix III, and therefore a deterministic subtraction circuit should be possible. However, this notion does not give us any clue on how to build one, how complex it may be, or what precision it can reach with a bounded complexity. Indeed, Liu and Parhi proposed a deterministic circuit for subtraction using one NOR gate ^22^ which yields very imprecise calculation unless ${p}_{1}\approx 1$ and ${p}_{0}\approx 0$, but can be improved by adding a series of "enhancement" circuits such that each additional circuit improves the precision. However, enhancement circuits are not "cheap": each involves two gates and one more flip-flop than the previous one, while precision is not much improved, even when so many enhancement circuits are used that the complexity exceeds one of the counter-based circuits. In particular, the subtraction becomes very imprecise as ${p}_{0}\to 1$. This is an example of a circuit whose error approaches zero (quite slowly) in the limit of infinite number of constituent gates.

**Figure 20:**
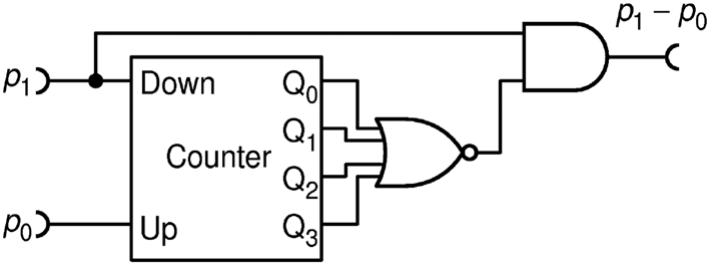
A simple and precise subtraction circuit SUB3 with counter of $N=4$ bits.

Towards finding a more economical and more precise solution, we, finally, propose a deterministic circuit SUB3 shown in Fig. 20, which exhibits significantly improved precision and a lower complexity than SUB2. Unfortunately, this circuit suffers a low relative KS entropy at the output.

**Figure 21:**
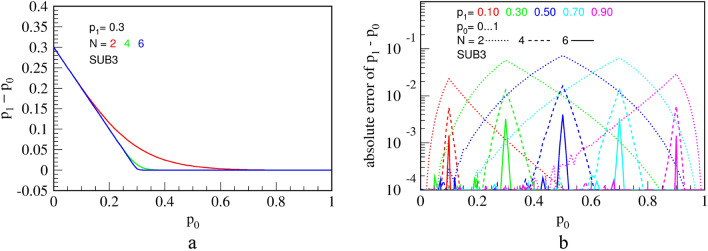
Measured transfer function **(a)**; and errors (difference from the ideal division) **(b)** for the circuit SUB3, for a fixed value of ${p}_{1}=0.3$ and counter capacities of $N=2, 4$ and $6$ bits.

It operates as follows. The counter memorizes how many pulses has arrived from ${p}_{0}$ and inhibits exactly that many pulses from ${p}_{1}$. Therefore, in principle, it should perform an exact subtraction. However, an error occurs if counter does not have enough capacity to count all pulses from ${p}_{0}$ before a pulse from ${p}_{1}$ arrives. The chance of this happening is highest when ${p}_{0}\approx {p}_{1}$ and can be lowered by using a bigger counter. Even though this subtractor is not derived from a divider, the transfer function and errors, shown in Fig. 21, resemble patterns seen in the division circuit DIV3 in Fig. 11, probably because both use counters in the feedback loop.

**Figure 22:**
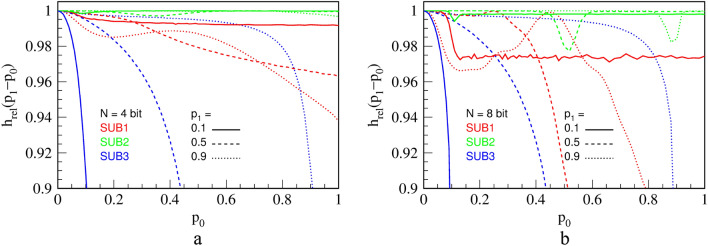
Relative KS entropy of the output of the three subtraction circuits that calculate ${p}_{1}-{p}_{0}$, for ${p}_{1}=$ 0.1, 0.5 and 0.9, for a counter of $N = 4$ bits **(a)** and $N = 8$ bits **(b)**.

A sharp edge between linear parts for $N\ge 4$ indicates small computation errors. In fact, explanation of the transfer function and errors is quite similar to that of the division circuit DIV3 in Fig. 11. The output RPT of SUB3 will consist of alternating parts of high and low frequency, a behavior similar to and of the same origin as explained for the division circuit DIV3. This leads to an entropy lower than maximal for a given output probability of pulses ${p}_{z}$. In addition, Fig. 22 shows relative KS entropy of measured outputs of the three presented subtraction circuits.

To conclude, we see that SUB2 has by far the best entropy for virtually any combination of ${p}_{0}$ and ${p}_{1}$, which is expected since it has been derived from DIV2. Interestingly, the deterministic circuit SUB3 performs better in terms of entropy than SUB2 for large values of ${p}_{1}$, as well as being far more precise. Contrarily, for smaller values of ${p}_{1}$, its entropy performance is much worse than that of the other two circuits for reasons explained above. As argued for DIV3, SUB3 can be used in favorable input conditions (${p}_{1}>0.9$) and is a circuit of choice for the last circuit in a calculation chain.

### A flow control circuit: the magnitude comparator

To complete the full set of circuits needed to build a universal computing machine, except having calculation circuits, a universal computing machine must have a flow control that allows for decision tree branching loops etc. This is usually done through a function that compares two numbers, such as ${p}_{0}>{p}_{1}$, which returns TRUE or FALSE (logical 1 or 0, respectively), while in the RPC it would return an RPT with $p=1$ or $p=0,$ respectively.

**Figure 23:**
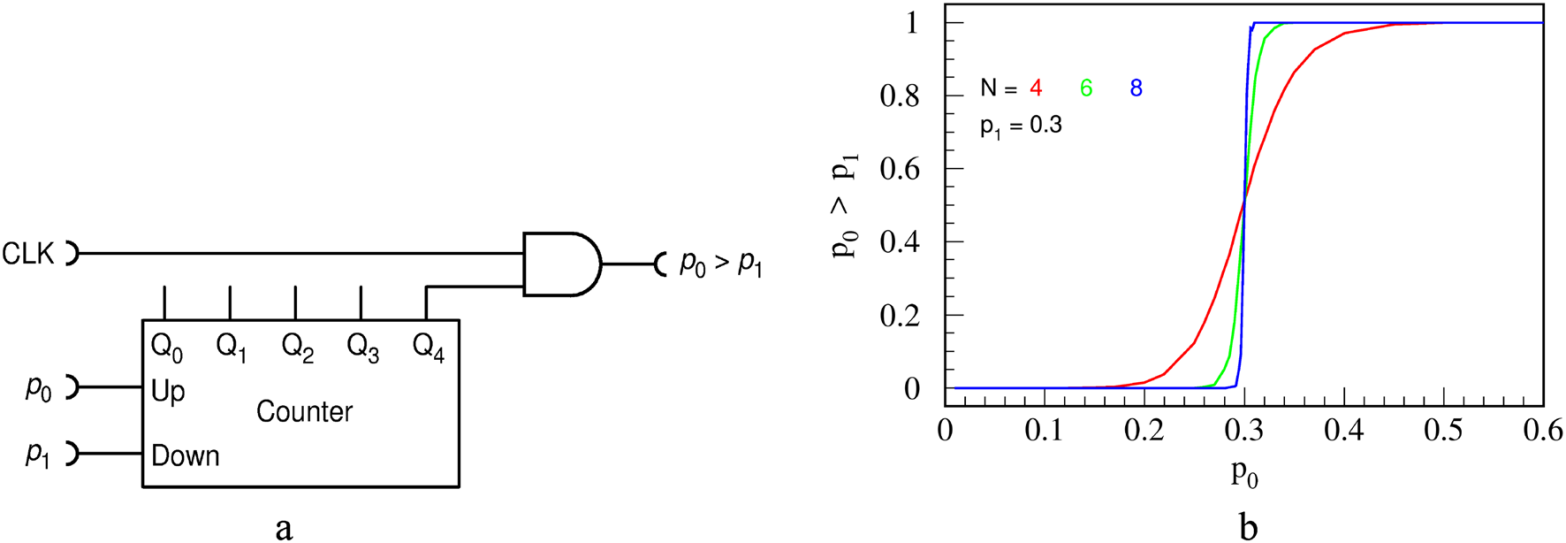
The comparator circuit **(a)** and its transfer functions for various counter bit-lengths **(b)**.

We note that subtraction circuit SUB3 goes into saturation and yields 0 for all ${p}_{0}>{p}_{1}$, which, in theory, could be used to detect that ${p}_{0}>{p}_{1}$. But this measure is not sharp: it will not switch to 1 as soon as ${p}_{0}<{p}_{1}$, rather, it would give a small value equal to ${p}_{1}-{p}_{0}$. Instead, we propose a simple circuit, shown in Fig. 23a, which performs an arbitrary sharp comparison function. Output, being an RPT rather than a logic state, means that it can assume a value anywhere in the range $\left[0, 1\right]$, not only exact value of 0 or 1.

We opted for 8-bit circuits as they are the most optimal, in our opinion, regarding highest precision and smallest amount of hardware used. This circuit yields 0 when ${{p}_{0}<p}_{1}$ and switch to 1 when ${p}_{0}>{p}_{1}$, with a sharpness that depends on the capacity of the counter, as seen in Fig. 23b. We find, heuristically, an approximate description of the transfer function of the circuit:

20 $${p}_{z}\left({p}_{0}, {p}_{1}\right)\approx \frac{1}{1+\mathrm{exp}\left({\frac{9}{8}2}^{N+1}\left({p}_{1}-{p}_{0}\right)\right)} .$$

The hardware efficiency of this circuit is best appreciated by noting that the slope (derivative) of the transfer function at the point ${p}_{0}={p}_{1}$ is equal to $\left(9/16\right){2}^{N}$, i.e., it rises exponentially with the length of the counter $N$.

If, instead of an RPT, a steady logic state (HIGH or LOW) is required, for example for interfacing to a conventional computer, then the AND gate and the CLK input can be omitted and the most significant bit (MSB) of the counter (in this case ${\mathrm{Q}}_{4}$) used as the output. Namely, the output probability ${p}_{z}\left({p}_{0}, {p}_{1}\right)$ equals the mean duty cycle of the MSB, thus state of the MSB is an optimal estimator of the state of the comparator circuit.

### Influence of non-maximal entropy to the precision of calculations

**Figure 24:**
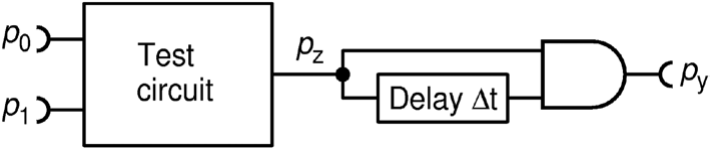
A circuit for testing an FPGA realization of a squaring circuit for a time-discrete RPT. The circuit multiplies the input RPT with its copy delayed by one period of the CLK clock.

In an RPC computer, complex functions are achieved by connecting basic operation circuits in a network. As noted, the crucial assumption for proper operation of an RPC circuit is that it receives pulse trains of maximal relative KS entropy, otherwise it may not function properly. To illustrate the problem, we use the standard squaring circuit^22^ fed by either division or subtraction circuit (DIV1-3, SUB1-3) that we studied above, as shown in Fig. 24.

This squaring circuit calculates square precisely when fed by an RPT. However, as already shown, circuits DIV1-3 and SUB1-3 produce PTs with a non-maximal relative KS entropy. To see how this influences precision of the computation, we define and measure two types of errors.

**Figure 25:**
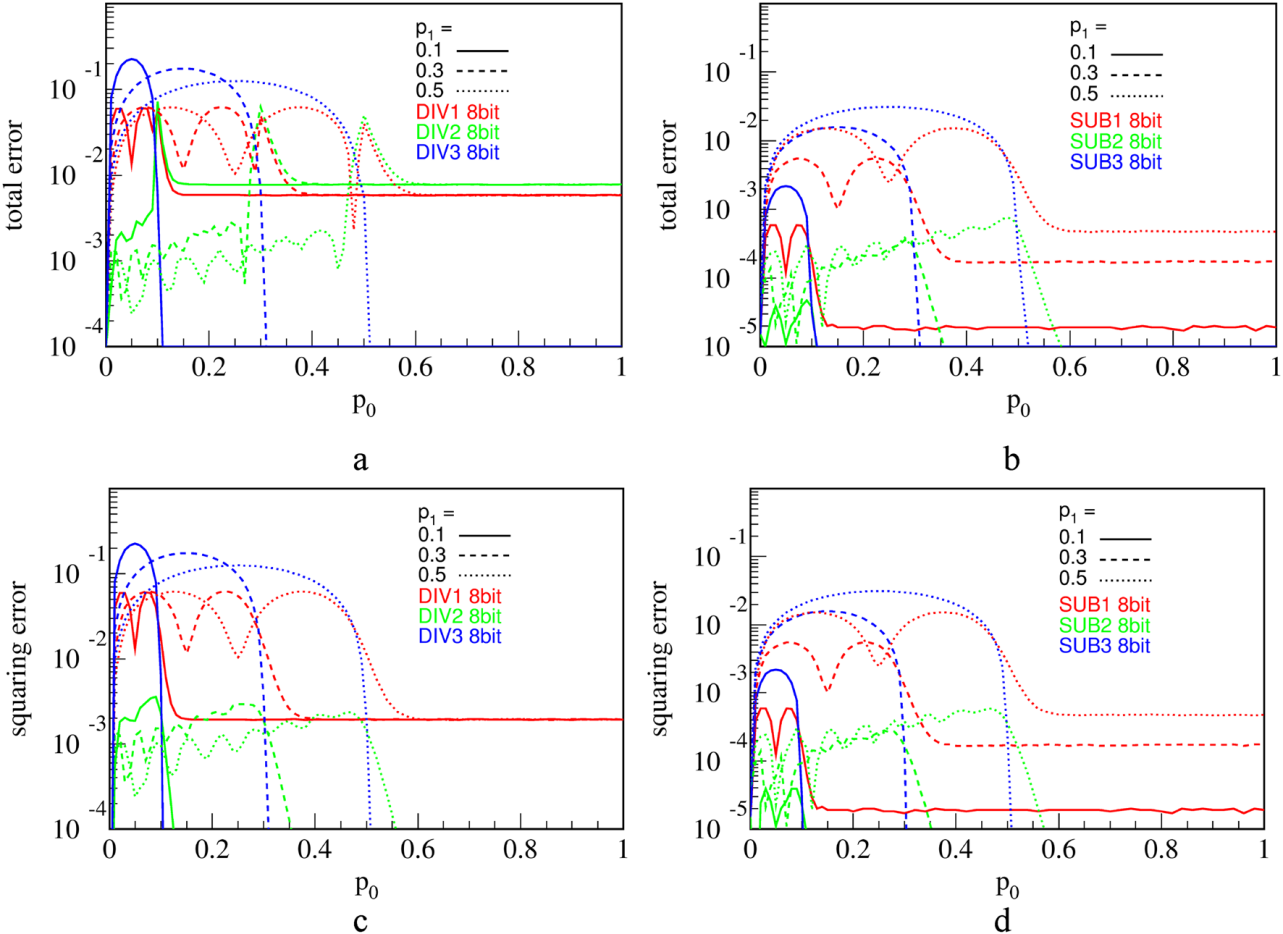
Measured computation errors for composite circuits: total errors of division and subtraction circuits followed by the square circuit **(a,b)**; squaring errors of division and subtraction circuits followed by the square circuit **(c,d)**. Statistical error margins on all figures are on the order of ${10}^{-6}$.

The first type of error is a total computation error, defined as ${p}_{y}-{\left({p}_{0}/{p}_{1}\right)}^{2}$ for a division circuit and ${p}_{y}-{\left({p}_{1}-{p}_{0}\right)}^{2}$ for a subtraction circuit. This error encompasses both errors of the DIV (SUB) circuits and the squaring operation. The respective results are shown in Fig. 25a,b. Referring to Figs. 15 and 22, we see that the superior output entropy performance of RFF-based circuits DIV2 and SUB2 translates into 1–2 orders of magnitude smaller error in the region of interest (where ${p}_{0}\le {p}_{1}$).

The second type of error accounts only for the error made by the squaring circuit, namely ${p}_{y}-{{p}_{z}}^{2}$, and is shown in Fig. 25c,d. This type of error shows how much the precision of the square circuit alone is affected by receiving the non-maximal input entropy. We emphasize that this error would be zero should inputs to the squaring circuit be RPTs. Instead, we find striking similarity of total error and squaring error curves in the region of interest for both DIV-Square and SUB-Square composite circuits. This means that the total error is dominated by the squaring error. The lower error is achieved by DIV1 and SUB1 circuits which yield 1–2 orders of magnitude smaller error than the other circuits, for any ${p}_{0}$ and ${p}_{1}$, because of their best performance in terms of the output entropy.

## Discussion and conclusions

Functional circuits for the RPC in a computing paradigm are a subject of vivid research. For the first time, we use notion of entropy in the study of RPC circuits. We find a rule, named "entropy budget criterion" (EBC), which states that output entropy of any physical RPC circuit is smaller or equal to the sum of all available input and internal entropies. Using the EBC, we find that binary and $n$-ary multiplication and subtraction, as well as half-addition, can be done by a deterministic circuit, while the other two arithmetic operations, namely addition and division, cannot be accomplished by deterministic circuits and require an additional source of entropy. By showing that deterministic half-addition and subtraction circuits are allowed by the EBC, their actual design becomes an open problem.

In the previous art, precision of RPC circuits has been analyzed under the assumption that their inputs are fed by perfect RPTs, while the level of randomness of their output pulse trains (PT) was ignored ^7–9^. This approach has led to a large calculation errors when circuits are chained, for example in evaluation of polynomials ^22^. In this study, we introduce relative KS entropy as a figure of merit randomness of PT, that is of closeness of a PT to an RPT with the same pulse probability. Based upon a case study of the squaring circuit, as well as theoretical arguments, we conclude that an RPC circuit, whose output PT has relative KS entropy less than 1, will generally cause a computational error in downstream circuits. Since, eventually, the goal is to build a universal RPC computer by networking RPC circuits, one should strive to design circuits with output relative KS entropy being as high as possible, while keeping the hardware cost of circuits as low as possible. Bearing in mind those two opposing requirements, we have presented here four new circuits (DIV2, DIV3, SUB2, SUB3) with improved output randomness and shown that they indeed cause lesser error in the subsequent calculation than the previously known ones. Finally, to build a programmable computer, one also needs a flow control circuit such as the comparator, which we presented in the last section.

In our search for novel RPC circuits, we use biologically-inspired approach. As it turns out, cell body of mammal neuron contains a specialized structure, the axon hillock ^36^, which processes signals from multitude of input synapses in a way similar to an up/down counter, followed by a comparator. On top of that, it features the ability to invert input signals and reset the counter. Excitatory pulses from synapses increase, while inhibitory pulses decrease the state of the neuronal counter. Literature is vague on how many pulses a neuron can count before going into saturation, but some works mention thousands^37^, which could account for up to 10–12 bits. Additionally, it has been found that some simple neurons emit random pulses without any input^38^. These may serve as a source of additional entropy needed to satisfy the EBC. Thus, live neurons integrate all of the functions required to build RPC circuits presented in this work.

Regarding hardware cost, a counter and a magnitude comparator each require dozens to hundreds of logic gates. There is clearly a dramatic rise in number of logic gates of the division and subtraction circuits, in comparison to the multiplication and addition circuits. On the other hand, low hardware cost is supposed to be one of the main advantages of the RPC. One approach is to build RPC circuits from sequential logic circuits only, as in the aforementioned work of Liu and Parhi^22^, which ended up with an overly complex subtraction circuit, which still has only a moderate precision.

Contrary to that approach, the biological neurons inspire us to follow the approach that includes counters, comparators, and random number generators. In doing so, one has to bear in mind that, while the digital approach, adopted here, is especially useful for understanding the basic principles of the RPC, it is not necessarily the most hardware-efficient way to realize them. Indeed, recent research has demonstrated neuron-like functions by the use of analog silicon chips^39^ or photon-induced plasticity of ${\mathrm{Si}}_{3}{\mathrm{N}}_{4}{\mathrm{SiO}}_{2}$ ^40^.

We believe that bio-inspired research of RPC circuits can lead to high-performance artificial networks as well as to better understanding of live neurons and live neuronal networks.

## Supplementary Information

Supplementary Information.
