# Supplementary material for: Entropy considerations in improved circuits for a biologically-inspired random pulse computer
Source: Sci Rep. 2022 Jan 7;12:115. doi: 10.1038/s41598-021-04177-9 (PMC8741937; doi:10.1038/s41598-021-04177-9)
Supplement: Supplementary file 1 — Supplementary Information. [file 41598_2021_4177_MOESM1_ESM.pdf]

Entropy considerations in improved circuits for a biologically-inspired random pulse computer

Mario Stipčević[1,*], Mateja Batelić[1,2]

[1]*Photonics and Quantum Optics, Center of Excellence for Advanced Materials and Sensing Devices, Ruđer Bošković Institute, Bijenička cesta 54, 10000 Zagreb, Croatia*
[2]*Department of Physics, Faculty of Science University of Zagreb, Bijenička cesta 32, 10000 Zagreb, Croatia*
**Corresponding author:* mario.stipcevic@irb.hr

APPENDIX I

Theorem 1. Let $\mathcal{H}(p) = -p\log_2 p - (1-p)\log_2(1-p)$ for $p \in [0,1]$ with a convention $\mathcal{H}(0) = \mathcal{H}(1) = 1$. Let $a, b \in [0,1]$. Then it is true:

$$\mathcal{H}(ab) \leq \mathcal{H}(a) + \mathcal{H}(b). \tag{1}$$

We first prove two lemmas.

Lemma 1.1. For $a, b \in (0, 1)$ holds the following inequality:

$$-ab\log_2 ab \leq -a\log_2 a - b\log_2 b\,. \tag{2}$$

Proof. Eq. (2) can be transformed to

$$-ab(\log_2 a + \log_2 b) \leq -a\log_2 a - b\log_2 b \tag{3}$$

$$-ab\log_2 a - ab\log_2 b \leq -a\log_2 a - b\log_2 b \tag{4}$$

The last holds true because all terms are non-negative and since $ab \leq a$ and $ab \leq b$, the inequality holds separately for first and second terms on both sides. Because of the range of a and b all logarithmic expressions are ≤ 0 thus Eq. (4) is equivalent to Eq. (2), Q.E.D.

Lemma 1.2. For $a, b \in (0, 1)$ holds the following inequality:

$$-(1-ab)\log_2(1-ab) \leq -(1-a)\log_2(1-a) - (1-b)\log_2(1-b). \tag{5}$$

Proof. We start from obvious inequality

$$(1-a)(1-b) \geq 0 \tag{6}$$

and add $(1-ab)$ to both sides

$$2 - a - b \geq 1 - ab \tag{7}$$

$$(1-a) + (1-b) \geq 1 - ab \tag{8}$$

Note that $(1-a)$, $(1-b)$ and $(1-ab)$ are all non-negative and therefore for any $x, y \geq 1$ it holds

$$(1-a)x + (1-b)y \geq 1 - ab. \tag{9}$$

Since $-\log_2(t)$ is a positive monotonically falling function on $(0,1)$and $(1-a) \leq (1-ab)$ it holds

$$\frac{\log_2(1-a)}{\log_2(1-ab)} \geq 1 \tag{10}$$

and the same holds for$-\log_2(1-b)$ in the nominator. We now define

$$x := \frac{\log_2(1-a)}{\log_2(1-ab)} \geq 1, \quad y := \frac{\log_2(1-b)}{\log_2(1-ab)} \geq 1 \tag{11}$$

and substitute for x and y in Eq. (9)

$$(1-a)\frac{\log_2(1-a)}{\log_2(1-ab)}+\frac{\log_2(1-b)}{\log_2(1-ab)}(1-b)\geq 1-ab. \qquad (12)$$

Multiplying both sides with a positive value $-\log_2(1-ab)$ we obtain

$$-(1-a)\log_2(1-a)-(1-a)\log_2(1-b)\geq -(1-ab)\log_2(1-ab). \qquad (13)$$

Q.E.D.

Proof of Theorem 1. Summation of the two lemmas yields Eq. (1) for $a, b \in (0,1)$. By analytical continuation and using definitions from Theorem 1, it is proven in the full interval $a, b \in [0,1]$.

APPENDIX II

Theorem 2. Let $\mathcal{H}(p) = -p\log_2 p-(1-p)\log_2(1-p)$ for $p \in [0,1]$ with a convention $\mathcal{H}(0)=\mathcal{H}(1)=1$. Let $a, b \in [0, 0.5]$. Then it is true:

$$\mathcal{H}(a+b)\leq \mathcal{H}(a)+\mathcal{H}(b)\,. \qquad (1)$$

We first prove two lemmas.

Lemma 2.1. For $a, b \in (0, 0.5)$ holds the following inequality:

$$-(a+b)\log_2(a+b)\leq -a\log_2(a)-b\log_2(b)\,. \qquad (2)$$

Proof. We first prove relation:

$$-a\log_2(a+b)\leq -a\log_2(a)\,. \qquad (3)$$

It holds:

$$a+b\geq a\,. \qquad (4)$$

Since $\log_2(t)$ is a monotonically increasing function on $(0, 0.5)$, it follows:

$$\log_2(a+b)\geq \log_2(a) \qquad (5)$$

and multiplying the above relation with $-a \leq 0$, we get:

$$-a\log_2(a+b)\leq -a\log_2(a)\,, \qquad (6)$$

which proves the relation (3).
Analogously, the relation

$$-b\log_2(a+b)\leq -b\log_2(b) \qquad (7)$$

holds true. Finally, summing the relations (3) and (7) yields Eq. (2), Q.E.D.

Lemma 2.2. For $a, b \in (0, 0.5)$ holds the following inequality:

$$-(1-(a+b))\log_2(1-(a+b))\leq -(1-a)\log_2(1-a)-(1-b)\log_2(1-b)\,. \qquad (8)$$

Proof. To prove Eq. (8), we prove the existence of the maximum of the following function:

$$f(a,b)=-(1-(a+b))\log_2(1-(a+b))+(1-a)\log_2(1-a)+(1-b)\log_2(1-b). \qquad (9)$$

The extreme point for this function is obtained by calculating the derivative over each variable a and b and equalizing with zero.

$$\frac{df(a,b)}{da}=\frac{\ln\big(1-(a+b)\big)-\ln(1-a)}{\ln(2)}=0 \;\Rightarrow\; b=0 \qquad (10)$$

$$\frac{df(a,b)}{db}=\frac{\ln\big(1-(a+b)\big)-\ln(1-b)}{\ln(2)}=0 \;\Rightarrow\; a=0 \qquad (11)$$

We see that if at least one variable is equal to zero, then function $f(a,b)$ acquires the zero value, which is the extreme value of this function. So, $f(0,b)=f(a,0)=f(0,0)=0$. Now, it only remains to see if this value is minimum or maximum value of the function. Since $-\log_2(t)$ is a monotonically decreasing function on $(0, 0.5)$ and function $f(a,b)$ can be rewritten as

$$f(a,b)=-\log_2\left(\frac{(1-(a+b))^{1-(a+b)}}{(1-a)^{1-a}(1-b)^{1-b}}\right), \tag{12}$$

it is plain to see that the function's maximum value is 0. This leads to $f(a,b)\ \leq 0$ so:

$$-(1-(a+b))\log_2(1-(a+b))+(1-a)\log_2(1-a)+(1-b)\log_2(1-b)\leq 0\,, \tag{13}$$

$$-(1-(a+b))\log_2(1-(a+b))\leq -(1-a)\log_2(1-a)-(1-b)\log_2(1-b)\,. \tag{14}$$

Q.E.D.

Proof of Theorem 2. Summation of lemmas 2.1. and 2.2. yields Eq. (1) for $a,b\in(0,0.5)$. By analytical continuation and using definitions from Theorem 2, this Theorem is proven in the full interval $a,b\in[0,0.5]$.

APPENDIX III

Theorem 3. Let $\mathcal{H}(p)=-p\log_2 p-(1-p)\log_2(1-p)$ for $p\in[0,1]$ with a convention $\mathcal{H}(0)=\mathcal{H}(1)=1$. Let $a,b\in(0,1)$ and $a\geq b$. Then it is true:

$$\mathcal{H}(a-b)\leq\mathcal{H}(a)+\mathcal{H}(b). \tag{1}$$

Proof. To prove this theorem, we see that $\mathcal{H}(x)=\ \mathcal{H}(1-x)$, $\forall x\in[0,1]$, so relation (1) can be rewritten as:

$$\mathcal{H}(1-(a-b))\leq\mathcal{H}(1-a)+\mathcal{H}(b) \tag{2}$$

$$\mathcal{H}((1-a)+b)\leq\mathcal{H}(1-a)+\mathcal{H}(b) \tag{3}$$

Since the function $\mathcal{H}(x)$ is concave because $\frac{d^2\mathcal{H}(x)}{dx^2}=\ -\frac{1}{x(1-x)\ln(2)}<0$ for $x\in\langle 0,1\rangle$, it holds for every $t,x\in[0,1]$:

$$\mathcal{H}(tx)=\mathcal{H}(tx+(1-t)\cdot 0)\geq t\mathcal{H}(x)+(1-t)\mathcal{H}(0)\geq t\mathcal{H}(x). \tag{4}$$

Now, let $x,y\in[0,1]$. Then, we have:

$$\mathcal{H}(x)+\mathcal{H}(y)=\ \mathcal{H}\left((x+y)\cdot\frac{x}{x+y}\right)+\mathcal{H}\left((x+y)\cdot\frac{y}{x+y}\right). \tag{5}$$

By applying inequality (4), we get:

$$\mathcal{H}(x)+\mathcal{H}(y)\geq\frac{x}{x+y}\mathcal{H}(x+y)+\ \frac{y}{x+y}\mathcal{H}(x+y) \tag{6}$$

$$\mathcal{H}(x)+\mathcal{H}(y)\geq\mathcal{H}(x+y) \tag{7}$$

This inequality obviously holds as it is proven in the Theorem 2. Finally, by defining $x\coloneqq 1-a$ and $y\coloneqq b$, Theorem 3 is proven in the full interval $a,b\in[0,1]$.
